# Human rhinovirus serotypes induces different immune responses

**DOI:** 10.1186/s12985-021-01701-1

**Published:** 2021-11-27

**Authors:** Ji Heui Kim, Jung Yeon Jang, Yong Ju Jang

**Affiliations:** Department of Otorhinolaryngology - Head and Neck Surgery, Asan Medical Center, University of Ulsan College of Medicine, 88 Olympic-ro 43-gil, Songpa-gu, Seoul, 05505 Republic of Korea

**Keywords:** Human rhinovirus, Interferon, IL-8, TLR3, MDA5, NF-κB, Cytokines

## Abstract

**Background:**

Different species of human rhinovirus (HRV) can induce varied antiviral and inflammatory responses in human blood macrophages and lower airway epithelium. Although human nasal epithelial cells (HNECs) are a primary infection route of HRV, differences between major and minor groups of HRV in the upper airway epithelium have not been studied in detail. In this study, we investigated viral replications and immune responses of major and minor groups of HRV in the HNECs.

**Methods:**

Viral replication, immune responses of IFN-β, IFN-λ, proinflammatory cytokines, and viral receptors, and mRNA expression of transcription factors of HRV16 (major group) and HRV1B (minor group) in the HNECs were assessed.

**Results:**

Compared with HRV16, HRV1B replicated more actively without excessive cell death and produced higher IFN-β, IFN-λ1/3, CXCL10, IL-6, IL-8, and IL-18 levels. Furthermore, low-density lipoprotein receptor (LDLR), TLR3, MDA5, NF-κB, STAT1, and STAT2 mRNA levels increased in HRV1B-infected HNECs.

**Conclusion:**

HRV1B induces a stronger antiviral and inflammatory response from cell entry to downstream signaling compared with HRV16.

**Supplementary Information:**

The online version contains supplementary material available at 10.1186/s12985-021-01701-1.

## Background

Human rhinovirus (HRV) is the most common cause of the common cold. Furthermore, HRV is detected in the nasal lavage and mucosae as well as turbinate epithelial cells of patients with chronic rhinosinusitis (CRS), suggesting a significant association between HRV infection and pathogenesis of CRS [1, 2]. HRV infections can also trigger severe lower airway diseases such as bronchitis, pneumonia, and exacerbations of asthma and chronic obstructive pulmonary disease [3–7].

HRVs are classified phylogenetically into three species (A, B, and C), including around 160 serotypes that differ in their surface proteins [8]. Different receptors between major and minor groups of HRV can elicit different immune and inflammatory responses. The major groups HRV-A and HRV-B enter the respiratory epithelial cells via Inter-Cellular Adhesion Molecule 1 (ICAM-1, CD54), whereas minor group HRV uses the low-density lipoprotein receptor (LDLR) [8]. HRV-C binds to cadherin-related family member 3 [9]. After their internalization via ICAM-1 or LDLR, the RNA genome of HRV crosses the endosome membrane into the cytosol [10]. In the endosome, viral double-stranded RNA (dsRNA) and single-stranded RNA (ssRNA) are recognized by toll-like receptor 3 (TLR3) and TLR7/8, respectively. Newly synthesized viral dsRNA and ssRNA in the cytoplasm are also recognized by retinoic acid-inducible gene I (RIG-I) and melanoma differentiation-associated gene 5 (MDA5). TLRs, RIG-1, and MDA-5 stimulate interferon-β (IFN-β) and INF-λ responses and production of proinflammatory cytokines and their gene expression, including C-X-C motif chemokine 10 (CXCL10), interleukin (IL)-6, and IL-8/CXCL8 [11, 12]. In a previous study, HRV16 (major group) and HRV1A (minor group) infections induced phosphorylation of kinases (p38, JNK, ERK5) and transcription factors (ATF-2, CREB, CEBP-alpha) differently in human macrophages derived from blood. Differential activation of these signaling pathways led to altered expression of inflammatory cytokines Chemokine (C–C motif) ligand 20 (CCL20), CCL2, and IL-10 [13].

Although human nasal epithelial cells (HNECs) are a primary infection route of HRV, there are no reports stating the differences in the inflammatory responses between major and minor groups of HRV in the HNECs. Minor group HRV1B can be infected in the lungs and sinonasal mucosa in Balb/c mice and induce airway inflammation [14, 15]. In our previous studies, it was identified that major group HRV16 induces inflammatory responses and alters tight and adherens junctions in HNECs, which may have deleterious effects on the barrier function of HNECs [16–18]. In addition, HRV16 infection up-regulates bacterial adhesion to HNECs, may induce secondary bacterial infections leading to bacterial rhinosinusitis [19–21]. In another previous study, HRV-A and HRV-B were detected in the nasal lavage fluid and turbinate epithelial cells of patients with CRS, and only HRV-A in the non-CRS controls [22]. Therefore, we focused on HRV1B (minor group of HRV-A) and HRV16 (major group of HRV-A) in the upper airway epithelium, and studied the immune responses elicited by both HRV16 and HRV1B infected cells, assessed the viral replications and measured the levels of IFN-β, IFN-λ, and proinflammatory cytokines produced by the infected cells.

## Methods

### Air–liquid interface (ALI) culture and virus infection in HNECs

This study was conducted in accordance with the Declaration of Helsinki and approved by the Institutional Review Board of Asan Medical Center (2017–0668). All participants provided written informed consent for inclusion before they participated in the study. HNECs were obtained from the middle turbinate of eight healthy subjects (4 males and 4 females, ages 19–49) who underwent septoplasty, according to a previous study [23]. They had no history of allergy and asthma, upper respiratory infection, and systemic or topical corticosteroid or antibiotic medications for 4 weeks before surgery. Briefly, passage-2 HNECs (3 × 10^5^ cells/well) were seeded in 0.5 ml of culture medium on Transwell clear culture inserts (24 mm, with a 0.4-μm pore size; Costar; Corning Inc., Corning, NY). Cells were cultured in a 1:1 mixture of basal epithelial growth medium and Dulbecco’s modified Eagle’s medium containing previously described supplements [23]. Cultures were grown in the submerged state for the first 9 days. The culture medium was changed on the first day, and thereafter, every alternate day. On day 9, the apical medium was removed to create an ALI, and cultures were supplied from the basal compartment only. All experiments described here used HNECs on day 14 after ALI creation, and their differentiation was confirmed by increased *TEKT1* and *MUC5AC* mRNA expression levels and decreased *SPRR1* mRNA levels and immunofluorescence staining for α-tubulin and MUC5AC.

HRV16 (major group) and HRV1B (minor group) (American Type Culture Collection, Manassas, VA) were grown and titered in HeLa cells as previously described [24]. HNECs were either mock-infected with phosphate buffered saline (PBS) or inoculated with HRV16 or HRV1B at a multiplicity of infection of 0.5 for 4 h at 33 °C, and washed with PBS. Cells were then incubated at 37 °C in 5% CO2 for 72 h.

### Real-time PCR

Total RNA was isolated from HNECs infected with HRV16 and HRV1B on the third day using RNeasy Mini Kit (Qiagen, Hilden, North Rhine-Westphalia, Germany) according to the manufacturer's instructions.

Viral RNA was quantified using an HRV16 or HRV1B Genesig® standard kit (Primerdesign™ Ltd., Chandler's Ford, UK). HRV RNA standards with pre-determined copy numbers included in the kit were used to generate standard curves for the quantification of HRV16 or HRV1B RNA. Real time-PCR was performed on an Applied Biosystems 7500 Fast Real-Time PCR instrument (Life Technologies Inc., Burlington, ON, Canada).

cDNA was synthesized using RT-&GO™ Mastermix (MP Biomedicals, Santa Ana, CA) according to the manufacturer’s protocol. Quantitative real-time PCR was performed using each cDNA with the Light-Cycler 480 SYBR Green I Master (Roche, Mannheim, Germany). The reaction mixture was prepared by the addition of a primer set to a LightCycler® real-time PCR system (Roche) following the manufacturer's instructions. PCR Primer sets for ICAM-1, LDLR, TLR3, TLR4, TLR7, TLR8, RIG-I, MDA5, signal transducer and activator of transcription 1 (STAT1), STAT2, nuclear factor kappa-light-chain-enhancer of activated B cells (NK-κB), IFN regulatory factor 3 (IRF3), IFR7, IRF9, and glyceraldehyde-3-phosphate dehydrogenase (GAPDH) are listed in Table 1. Reaction conditions were as follows: initial denaturation at 95 °C for 5 min, followed by 45 cycles of denaturation at 95 °C for 10 s, annealing at 55 °C for 20 s, and extension at 72 °C for 10 s. Melting temperature analysis was also performed (65–95 °C at 0.5 °C increments for reading the fluorescent signals). To analyze the data, we used LightCycler® 480 software, Version 1.5 (Roche). The results were normalized to GAPDH expression, and the relative gene expression was calculated using the comparative 2^−ΔΔCT^ method.

**Table 1:** Primer sequences used real-time PCR

Genes	Primer sequences
ICAM-1	Forward 5′-GCAGACAGTGACCATCTACAGCTT-3′ Reverse 5′-CTTCTGAGACCTCTGGCTTCGT-3′
LDL-R	Forward 5′-GACGTGGCGTGAACATCTC-3′ Reverse 5′-CTGGCAGGCAATGCTTTGG-3′
TLR3	Forward 5′-TTTGCGAAGAGGAATGTTTAAATCT-3′ Reverse 5′-CACCTATCCGTTCTTTCTGAACTG-3′
RIG-1	Forward 5′-GCTGATGAAGGCATTGACATTG-3′ Reverse 5′-CAGCATTACTAGTCAGAAGGAAGCA-3′
MDA-5	Forward 5′-CCCATGACACAGAATGAACAAAA-3′ Reverse 5′-CGAGACCATAACGGATAACAATGT-3′
NK-κB	Forward 5′-CCATGACAGCAAATCTCC-3′ Reverse 5′- TAAACTTCATCTCCACCCC-3′
IRF3	Forward 5′-TCTTCCAGCAGACCATCTCC-3′ reverse 5′-TGCCTCACGTAGCTCATCAC-3′
IRF7	Forward 5′-TACCATCTACCTGGGCTTCG-3′ Reverse 5′-TGCTGCTATCCAGGGAAGAC-3′
STAT1	Forward 5′-ATCAGGCTCAGTCGGGGAAT-3′ Reverse 5′-TGGTCTCGTGTTCTCTGTTCTGC-3′
STAT2	Forward 5′-GCCCTAGTTCCAGCTCTAATG-3′ Reverse 5′- CAGGCTCATTGTGGTCTCTAAT-3′
IRF9	Forward 5′-CCCGAAAACTCCGGAACTG-3′ Reverse 5′-CAGCACACTCCGGGAAACT-3′
GAPDH	Forward 5′-GACCCCTTCATTGACCTC-3′ Reverse 5′-GCTAAGCAGTTGGTGGTG-3′

### Measurement of lactate dehydrogenase

Lactate dehydrogenase (LDH) activity was measured to evaluate degree of cell death caused by viral infection using the LDH assay kit (BioVision, Mountain View, CA, USA) according to the manufacturer’s protocol.

### Immunofluorescence staining

Fixed and permeabilized cells were incubated with diluted mabJ2 in PBS/1% BSA (0.33 μg/ml, which corresponded to a 1:1500 dilution of the antibody at 0.5 mg/ml) at room temperature for 1 h. Cells were washed twice with PBS. The R16-7 monoclonal antibody (mAb) (QED Bioscience Inc., San Diego, CA) recognizes the capsid protein VP2 and its precursor VP0 and P1 of RV16. R16-7 mAb at 10 μg/ml and anti-serum HRV1B at 5 μg/ml were incubated at 4 °C overnight. Antiserum against HRV1B was raised to HRV1B-infected HeLa whole cell lysates. The antiserum was partially purified by incubating with nitrocellulose-bound HeLa cell proteins. The cells were then incubated with secondary antibody Alexa Fluor® 488 donkey anti-mouse IgG (20 μg/ml; Abcam, Cambridge, UK) to R16-7 mAb and Alexa Fluor® 488 goat anti-guinea pig IgG (7 μg/ml; Invitrogen, Carlsbad, CA, USA) to anti-serum HRV1B for 60 min at 24 °C in the dark. Controls were incubated with the same concentrations of mouse IgG isotype control (Invitrogen) and guinea pig IgG isotype control (Invitrogen). Cells were stained with 4,6-diamidino-2-phenylindole (DAPI; Invitrogen), mounted with proLong antifade medium (Invitrogen), and examined using a confocal laser scanning microscope (ZEISS LSM 780; Carl Zeiss Microscopy GmbH, Jena, Germany). The fluorescence intensity of Alexa Fluor® 488 was quantified by ImageJ (National Health Institute, Bethesda, MA, USA).

### ELISA

IFN-β, IFN-λ1/3, CXCL10, IL-6, IL-8, and IL-18 concentrations in the basal supernatants were measured with a human DuoSet ELISA kit (R&D Systems, Minneapolis, MN, USA) according to the manufacturer’s instructions.

### Statistical analysis

The variables were compared using a paired Wilcoxon signed-rank test. The significance of a correlation was assessed using the Pearson’s correlation test. Data were analyzed using GraphPad Prism v.9.00 (GraphPad Software Inc.). Statistical significance was defined at *P* < 0.05.

## Results

### HRV16 and HRV1B replication

Initially, we checked the viral RNA and LDH activity after HRV16 infection in A549 cell lines, which is a human epithelial cell line derived from a lung carcinoma tissue, at 24, 48, 72, and 96 h incubated at 33 °C and 37 °C after infection for 4 h at 33 °C. HRV16 RNA was highest at 24 h post-infection and decreased slightly, but remained until 72 h post-infection without statistical significance. There was no significant difference in HRV16 RNA between 33 °C and 37 °C incubation temperature. However, LDH activity was significantly increased in incubated cells at 33 °C from 24 to 96 h, but not significantly different between 24, 48, 72, and 96 h post-infection in incubated cells at 37 °C (Additional file 1: Fig. S1a). The IFN-λ1/3 and IL-6 levels consistently increased from 24 to 72 h and remained unchanged from 72 to 96 h (Additional file 1: Fig. S1b). Hence, we focused on the immune response to HRV infections in HNECs at 72 h and determined 37 °C of incubation temperature considering the significantly increased cytotoxicity at 33 °C.

In addition, to determine the viral load, submerged primary nasal epithelial cells were infected with 0.5 and 1 MOI HRV16, and HRV16 RNA at 72 h and LDH activity and IFN-β, IFN-λ1/3, IL-6 levels after HRV16 infection were measured at 24, 48, and 72 h post-infection (Additional file 1: Fig. S2). HRV RNA, LDH activity, and IFN-β levels were not different between cells infected with 0.5 MOI and cells infected with 1 MOI in all time points. Although IFN-λ1/3 and IL-6 levels were higher in cells in cells infected with 0.5 MOI or 1 MOI compared to uninfected cells at each time point, there were no differences between cells infected with 0.5 MOI and cells infected with 1 MOI in all time points. Therefore, 0.5 MOI of HRV was determined considering viral replication, cell cytotoxicity, and cytokine production.

**Fig. 1:**
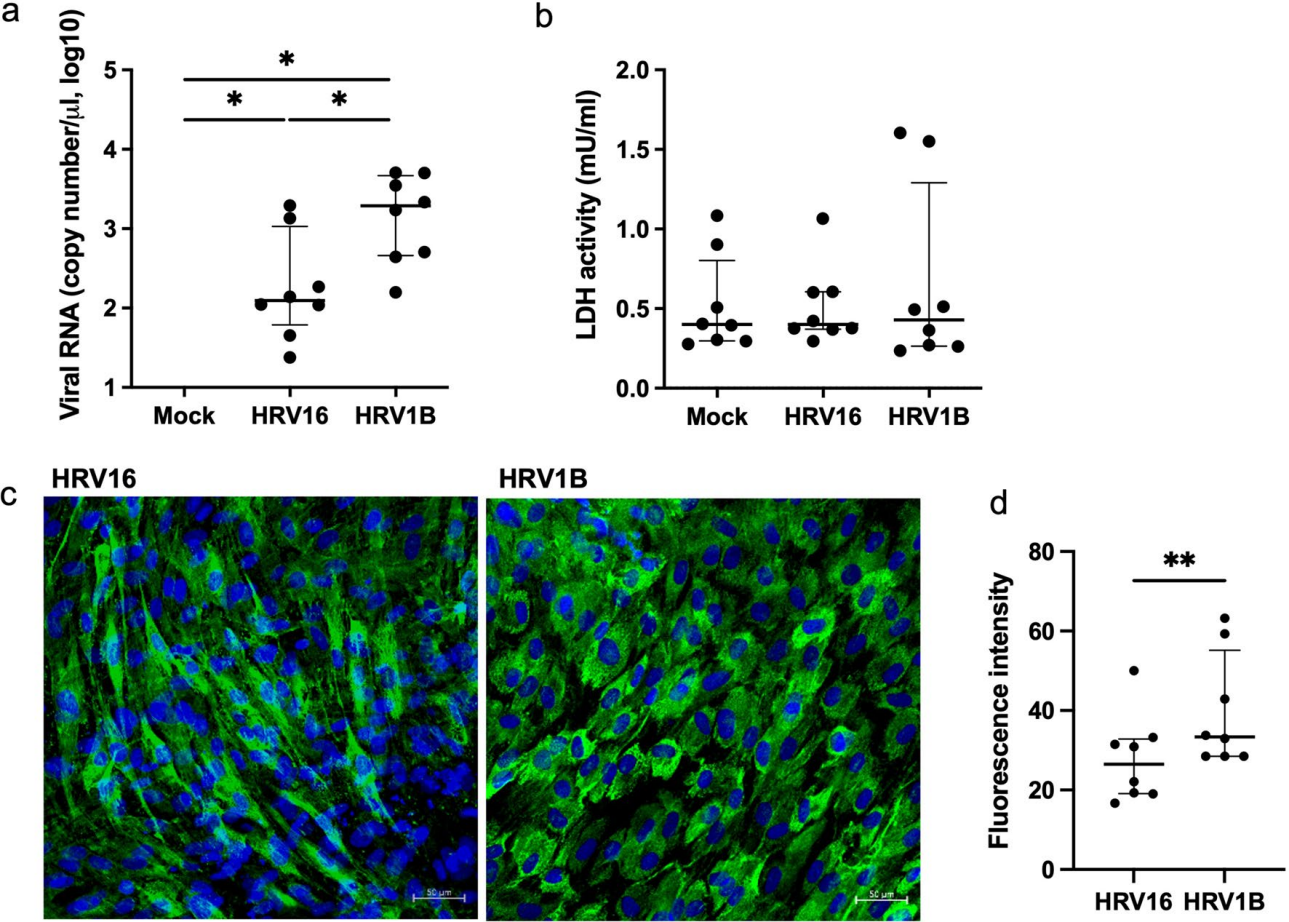
Viral RNA in HRV16 and HRV1B-infected HNECs at 72 h. **a** Copy number of HRV1B RNA was higher than that of HRV16 RNA. **b** LDH activity in HRV16 and HRV1B infected HNECs were not different with that in mock-infected HNECs. **c** HRV16 and HRV1B RNA were detected in the cytoplasm of HNECs. HRV1B were more abundant than HRV16 (× 200 magnification, scale bar: 50 μm). **d** Fluorescence intensity was higher in HRV1B than in HRV16. Results are presented as the median with and interquartile range from eight subjects.*p < 0.05, ** *p* < 0.01 in Wilcoxon signed-rank test

At 72 h, HRV16 and HRV1B RNA in the infected cells increased (*p* = 0.008 and *p* = 0.012, respectively) compared to that of the mock; the replication of HRV1B RNA was higher than that of HRV16 (*p* = 0.016) (Fig. 1a). LDH activity remained similar across the mock and HRV16 infections, while showing a slight increase in HRV1B infections (all *p* > 0.05) (Fig. 1b). Immunofluorescence staining revealed that HRV1B was more abundant in the cytoplasm of HNECs than HRV16, and fluorescence intensity was higher in HRV1B than in HRV16 (*p* = 0.008) (Fig. 1c, d).

### Interferon and proinflammatory cytokine production

**Fig. 2:**
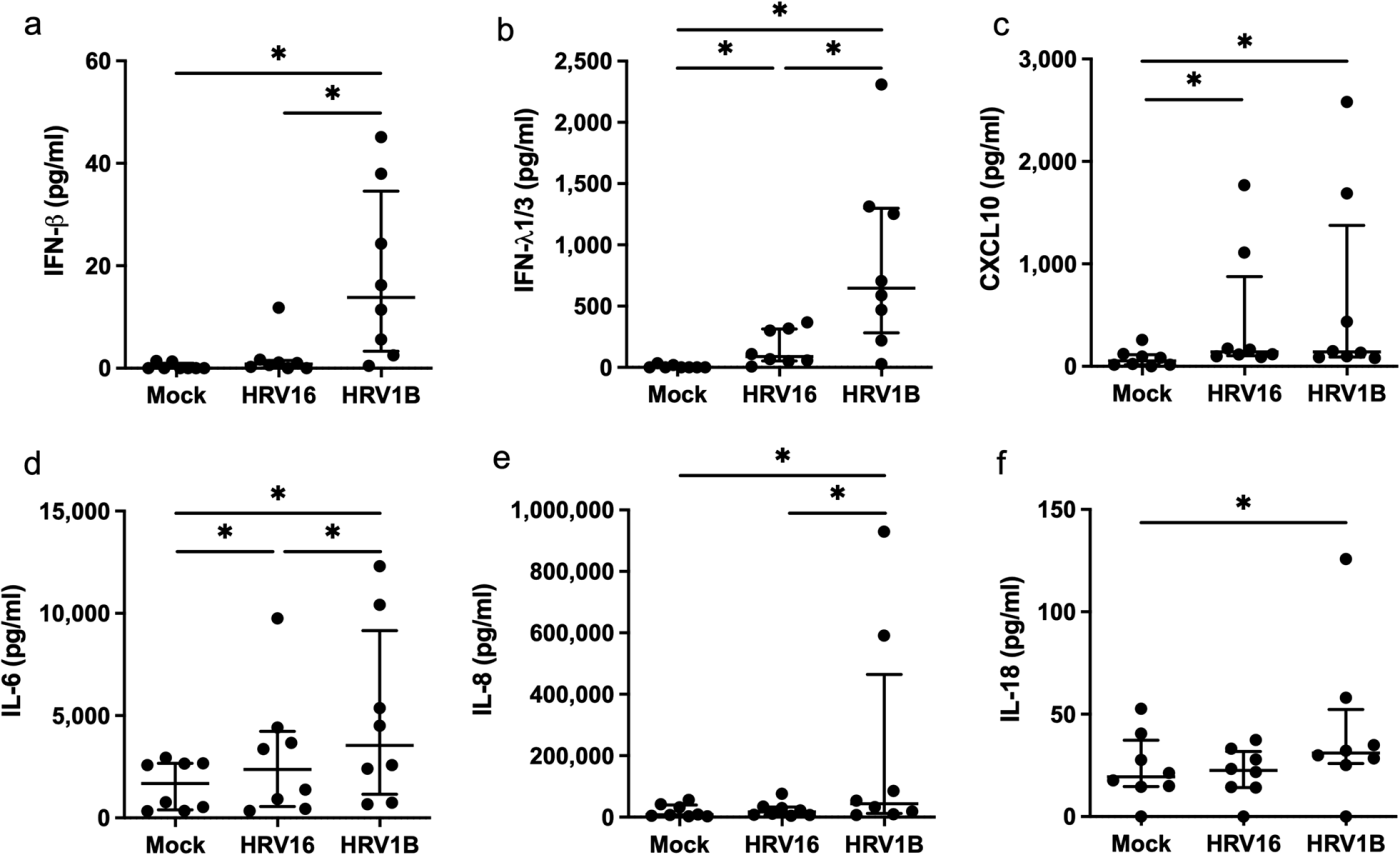
Interferons and proinflammatory cytokine production in HRV16 and HRV1B-infected HNECs at 72 h. (**a**-**f**) IFN-β, IFN-λ1/3, CXCL-10, IL-6, IL-8, and IL-18 production were significantly increased in HRV1B-infected HNECs compared to uninfected HNECs. (**b**-**d**) IFN-λ1/3, CXCL-10, and IL-6 levels were significantly higher in HRV16-infected HNECs than those in uninfected HNECs. IFN-λ1/3 and IL-6 levels were higher in HRV1B-infected HNECs than those in HRV16-infected HNECs. Results are presented as the median with and interquartile range from eight subjects. *p < 0.05 in Wilcoxon signed-rank test

IFN-β production significantly increased in HRV1B infection (*p* = 0.028), but not in HRV16 infection (*p* = 0.465), whereas IFN-λ1/3 production increased in both HRV16 and HRV1B infections (*p* = 0.011 and *p* = 0.017, respectively). IFN-β and IFN-λ1/3 levels in HRV1B-infected cells were higher than those in HRV16-infected cells (*p* = 0.043 and *p* = 0.028, respectively) (Fig. 2a, b).

The production of proinflammatory cytokines CXCL10 and IL-6 increased in both HRV16 and HRV1B infection (*p* < 0.05 for each), whereas IL-8 and IL-18 production increased in HRV1B infection (*p* = 0.025 and *p* = 0.018, respectively), but not in HRV16 infection (*p* = 0.314 and *p* = 0.674, respectively). IL-6 and IL-8 levels in HRV1B were higher than HRV16 levels (*p* = 0.018 and *p* = 0.018, respectively) (Fig. 2c–f).

**Fig. 3:**
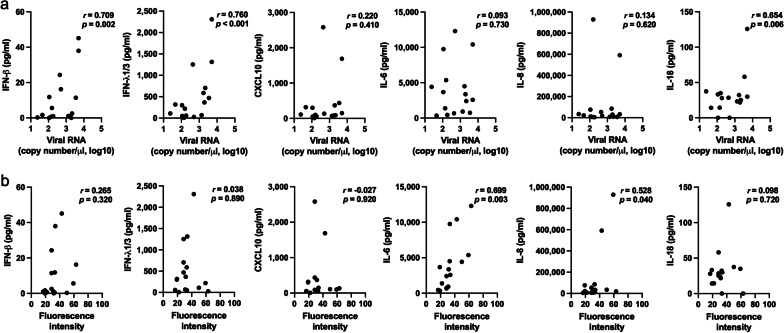
Correlation between viral RNA (**a**) and fluorescence intensity of HRV (**b**) and interferons and proinflammatory cytokine levels in HRV-infected HNECs assessed using Pearson’s correlation test

IFN-β, IFN-λ1/3, and IL-18 levels positively correlated with HRV RNA copy number (*p* = 0.002, *p* < 0.001, and *p* = 0.006, respectively), and IL-6 and IL-8 levels positively correlated with the fluorescence intensity of HRV (*p* = 0.003 and *p* = 0.040, respectively) (Fig. 3a, b).

### TLR3 and MDA5 mRNA expression

**Fig. 4:**
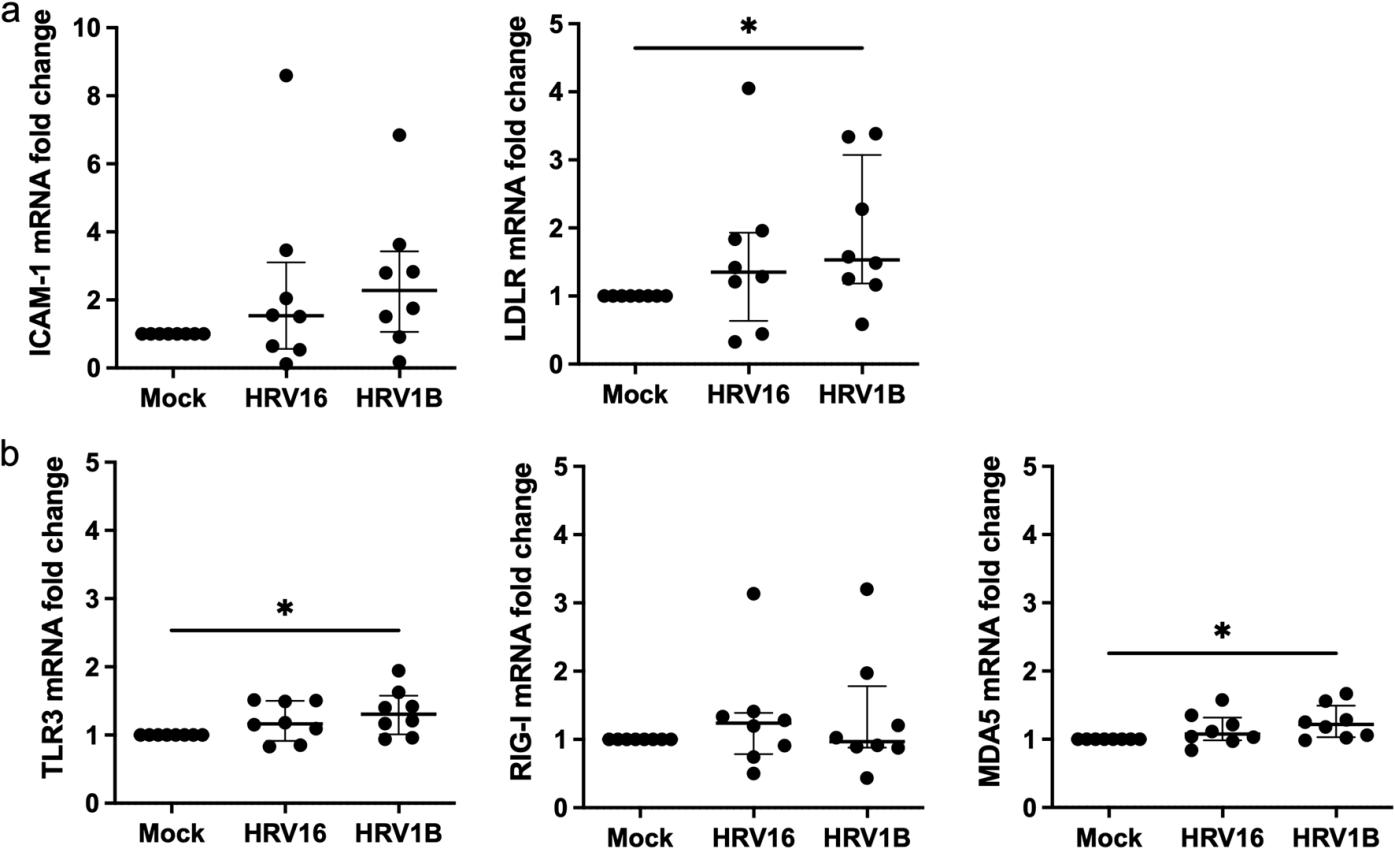
mRNA expression levels of cellular receptors and pattern recognition receptors. (**a**) There was no statistical significance in ICAM-1 mRNA levels between mock, HRV16, and HRV1B-infected HNECs. However, LDLR mRNA expression levels significantly increased in HRV1B-infected HNECs. (**b**) TLR3 and MDA5 mRNA levels were significantly increased in HRV1B-infected HNECs. Results are presented as the median with and interquartile range from eight subjects. *p < 0.05 in Wilcoxon signed-rank test

Although ICAM-1 mRNA levels increased in HRV16 and HRV1B-infected HNECs, there was no statistical significance between them (*p* = 0.093 and *p* = 0.069, respectively). LDLR mRNA expression levels significantly increased in HRV1B infections (*p* = 0.036) (Fig. 4a).

TLR3 and MDA5 mRNA levels were significantly higher in HRV1B infection (*p* = 0.036 and *p* = 0.017, respectively), but not in HRV16 infection (*p* = 0.093 and *p* = 0.069, respectively). The increase in RIG-I mRNA levels in both HRV16 and HRV1B infected cells was not significant (*p* = 0.327 and *p* = 0.674, respectively) (Fig. 4b). However, TLR3, RIG-I, and MDA5 mRNA levels were similar among themselves in HRV16 and HRV1B infected cells.

### Transcription factors and their mRNA expression

**Fig. 5:**
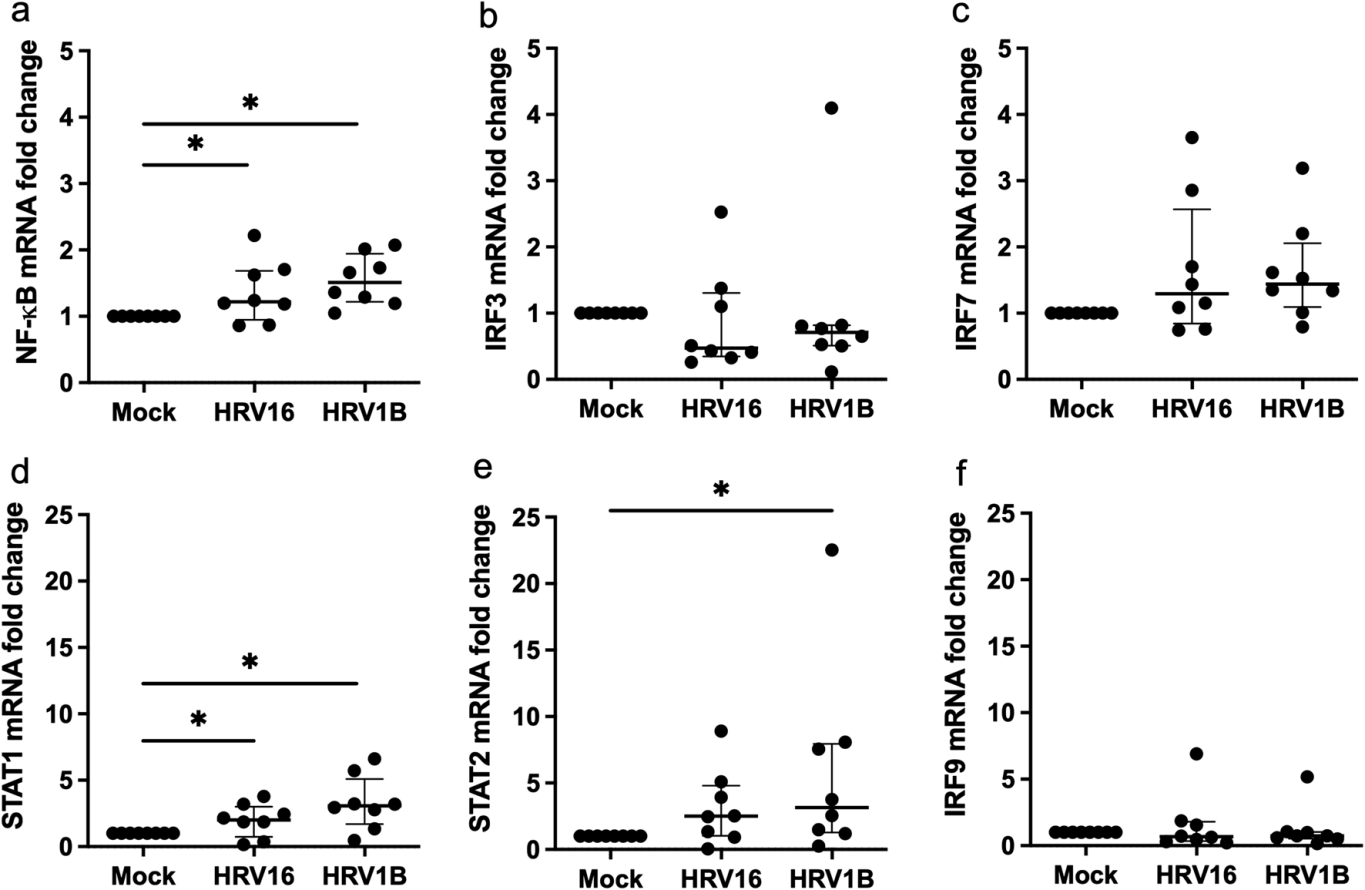
mRNA expression levels of transcription factors. NF-κB, STAT1, and STAT2 mRNA levels were increased in HRV1B-infected HNECs. (**a**-**f**) NF-κB, STAT1, and STAT2 mRNA levels were significantly increased in HRV1B-infected HNECs compared to uninfected HNECs. NF-κB and STAT1 mRNA levels were significantly increased in HRV16-infected HNECs compared to uninfected HNECs. However, IRF3, IRF7, and IRF9 mRNA levels were not increased in HRV16 and HRV1B infection. Results are presented as the median with and interquartile range from eight subjects. *p < 0.05 in Wilcoxon signed-rank test

NF-κB mRNA levels were significantly higher in HRV16 and HRV1B infection than in mock (*p* = 0.036 and *p* = 0.012, respectively) (Fig. 5a). IRF3 and IRF7 mRNA levels in HRV16 and HRV1B infection were not different from the mock (Fig. 5b, c). STAT1 mRNA levels were increased in both HRV16 and HRV1B infection than mock (*p* = 0.036 and *p* = 0.014, respectively) whereas STAT2 mRNA levels were increased in HRV1B infection, not in HRV16, than mock (*p* = 0.040) (Fig. 5d, e), whereas IRF9 mRNA levels were not increased in HRV16 and HRV1B infection (Fig. 5f).

## Discussion

In this study, HRV1B (minor group of HRV-A) induced stronger immune responses than HRV16 (major group of HRV-A) in the upper airway epithelial cells with more active replication of its viral RNA and increased production of IFN-β, IFN-λ1/3, and proinflammatory cytokines.

A previous in vitro study reported differences in immune responses between HRV serotypes in Calu-3 cells, a human lung cancer cell line. HRV14- (species B) and HRV16 (species A)-infected cells showed an increase in IL-6, basic fibroblast growth factor, and IFN-γ-induced protein 10. However, unlike HRV16 cells, HRV14-infected cells showed an increase in macrophage inflammatory protein (MIP)-1β, IFN-λ2/IL-28A, monocyte chemoattractant protein (MCP)-2, and IFN-α. The addition of peripheral blood mononuclear cells did not increase MIP-1β, IFN-λ2/IL-28A, and IFN-α levels in HRV16-infected Calu-3 cells, suggesting that altered regulation of cytokines might be a reason for variation in disease severity associated with different HRVs [25]. In our study’s ALI culture of upper airway epithelial cells, HRV16-infected cells, showed increase in the cytokines CXCL10, IL-6, and IFN-λ1/3 levels but no increase in IFN-β, IL-8, and IL-18, whereas HRV1B-infected cells showed an increase in IFN-β, IFN-λ1/3, CXCL10, IL-6, IL-8, and IL-18 production. Viral RNA and abundance of HRV in the cytoplasm were also higher in HRV1B-infected HNECs than those infected with HRV16. Moreover, IFN-β, IFN-λ1/3, and IL-18 levels positively correlated with viral RNA and IL-6 and IL-8 levels correlated with fluorescence intensity of HRV, which suggests that differences in viral replication and proliferation lead to the differences in immune factors production. This difference in viral replication may be caused by differences in the entry of HRV16 and HRV1B into cells, which is reflected by a non-significant increase in ICAM-1 mRNA expression in HRV16-infected HNECs, but a significant increase in LDL-R mRNA expression in HRV1B-infected HNECs. Moreover, TLR3 and MDA5 mRNA levels were higher in HRV1B-infected HNECs, but not in HRV16-infected cells. RIG-I mRNA levels were similar in both HRV16 and HRV1B-infected HNECS. Given that TLR3 and MDA5, but not RIG-I, are required for maximal sensing of RV dsRNA and type I IFN signaling pathway [26], the difference in IFN-β production between HRV16 and HRV1B-infected HNECs may account for the difference in TLR and MDA5 expression.

Nevertheless, our results may not be broadly applied to other serotypes in HRV-A-major and -minor groups. In a previous study, they compared immune responses between HRV-A55 (major group) and HRV-A49 (minor group) in addition to other highly prevalent respiratory pathogens such as HRV-B, HRV-C, respiratory enteroviruses, influenza virus, respiratory syncytial viruses, and coronaviruses [27]. There were no differences in viral load and chemokine and cytokine levels between HRV-A55 and HRV-A49 in contrast to our result that showed differences between HRV-16 and HRV-1B. These discrepancies may be due to differences in cell source, viral load, and location of sample collection. In our study, nasal epithelial cells from the middle turbinate tissue were used and infected with 0.5 MOI of virus (1.5 × 10^5^), whereas those from nasal polyps were used and infected with a larger load of 1.5 × 10^6^ in their study. Furthermore, viral load was quantified in the cells in our study, while those were measured released viruses to apical and basal medium in their study. However, IL-6 and IL-8 levels in the basal medium were different between HRV16 and HRV1B in our study, but not different between HRV-A55 and HRV-A49 in their study. Another study showed the differences in IL-8 and IFN-β levels between HRV16 and HRV1B [28]. Therefore, the immune response of HRV may be serotype specific.

IFNs are an important component of the antiviral innate immune response. Many cell types produce both type I IFNs (IFN-α/β) and type III IFN (IFN-λ) in response to a respiratory virus infection, and airway epithelial cells also produce IFN-β and IFN-λ. However, the amount and type of IFNs produced during viral infection depend on the cell types. Airway epithelial cells produce abundant IFN-λ in response to respiratory syncytial virus and influenza virus as well as poly I:C (a TLR-3 agonist), whereas IFN-β production was less than IFN-λ. [23, 29, 30] Likewise, IFN-λ was significantly produced in HNECs in response to HRV infection, while IFN-β was less produced than IFN-λ. CD11c + cells may be the source of the sinonasal mucosa [31].

In addition to IFNs, IL-18 is a major proinflammatory cytokine released upon activation of pattern recognition receptors and has broad pathogen-neutralizing effects, enhancing type 1 responses, activating neutrophils, and thereby promoting early innate immune responses [32]. Human bronchial epithelial cells can produce IL-18 in response to HRV infection [33], in our study, HNECs also produce IL-18, which is expected to prime neutrophils and enhance type 1 immune response to eradicate HRV.

NF-κB regulates the expression of many genes, including those of cytokines, chemokines, transcription factors, antimicrobial peptides, and IFN-stimulated genes [34–38]. IL-6 and IL-8 production in HRV-infected epithelial cells is mediated by the NF-κB-dependent transcriptional activation pathway [11]. In our study, NF-κB levels increased, both in HRV16 and HRV1B-infected HNECs. However, the increase in IRF3 and IRF7 mRNA levels after HRV infection was not significant. These findings suggest that NF-κB may be involved in types I and III IFN as well as proinflammatory cytokine production.

In the downstream signaling pathway of type I and III IFNs, Janus activated kinase engaged with IFN receptor phosphorylates both STAT1 and STAT2, leading to the formation of IFN-stimulated gene factor 3 complexes (STAT1-STAT2-IRF9 complexes). These complexes translocate to the nucleus and bind to IFN-stimulated response elements to transcribe IFN-stimulated genes [39]. Compared with HRV16-infected HNECs, a more prominent increase in STAT1 and STAT2 mRNA together with IFN-β and IFN-λ1/3 in HRV1B-infected HNECs may reflect stronger inflammatory and anti-viral immune responses.

Clinically, compared with the human rhinovirus species HRV-B, HRV-A, and HRV-C have been more strongly associated with moderate to severe illness in infants, and hospitalization for acute respiratory illness and serious illness outcomes in young children [40, 41]. However, while minor group HRV-A exposure was significantly associated with asthma exacerbations in adults, major group HRV-A, HRV-B, HRV-C, and other viruses were not. In addition, neutrophil burden was associated with asthma exacerbations [42]. IL-8, a neutrophil chemokine and activator, is an important factor in the clinical outcome of HRV infection [11]. Levels of IL-8 in nasal lavage fluid from HRV-infected subjects correlated with severity of rhinorrhea and nasal obstruction and peaked at 48 to 72 h after HRV inoculation [43]. Our study showed that IL-8 production obviously increased in HRV1B-infected HNECs at 72 h and was higher than IL-8 levels in HRV16-infected HNCEs, suggesting that upper respiratory infection symptoms (similar to asthma exacerbation) may be more severe in HRV1B infection than in HRV16 infection related to neutrophil burden [42]. Based on our results, it is important to find the identity of the HRV species or sequence elements that confer the most pathogenic risk. Further studies are needed regarding the mechanisms that affect antiviral responses and/or induce damage to the airway mucosa associated with neutrophil influx in patients with underlying airway disease, including asthma, COPD, and CRS.

## Conclusion

Our study showed that HRV1B (minor group of HRV-A) replicated more actively and induced increased production of IFN-β, IFN-λ1/3, CXCL10, IL-6, IL-8, and IL-18 in HNECs, compared with HRV16 (major group of HRV-A). Moreover, higher levels of LDL-R, TLR3, MDA5, NF-κB, STAT1, and STAT2 mRNA expression were seen in HRV1B-infected HNECs. These results suggest that selection of the serotype of HRV is very important when designing experiments to identify the immune response to HRV.

## Supplementary Information

**Additional file 1**.** Figure S1.** HRV16 RNA, LDH activity, and INF-λ1/3 and IL-6 levels after HRV16 infection in A549 cells. (a) The copy number of HRV16 was not different between 33°C and 37°C at 24 – 96 h post-infection. LDH activity also did not differ between 24, 48, 72, and 96 h post-infection. (c) INF-λ1/3 and IL-6 levels increased consistently from 24 to 72 h and remained unchanged from 72 to 96 h.** Figure S2.** Dose-response of HRV16 RNA, LDH activity, and IFN-β, IFN-λ1/3, and IL-6 production from submerged primary nasal epithelial cells after HRV16 infection. (a) At 72 h post-infection, HRV16 RNA was not different in nasal epithelial cells infected with 0.5 MOI and 1 MOI of HRV16. (b) At 24, 48, and 72 h post-infection, LDH activity and IFN-β, IFN-λ1/3, and IL-6 production were not different in nasal epithelial cells infected with 0.5 MOI and 1 MOI of HRV16.

## Data Availability

Available from the corresponding author, upon reasonable request.

## Abbreviations

HRV: Human rhinovirus
CRS: Chronic rhinosinusitis
ICAM-1: Inter-Cellular Adhesion Molecule 1
LDLR: Low-density lipoprotein receptor
TLR3: Toll-like receptor 3
RIG-I: Retinoic acid-inducible gene I
MDA5: Melanoma differentiation-associated gene 5
IFN: Interferon
CXCL: C-X-C motif chemokine
IL: Interleukin
CCL: Cytokines Chemokine (C–C motif) ligand
HNECs: Human nasal epithelial cells
ALI: Air–liquid Interface
PBS: Phosphate buffered saline
STAT: Signal transducer and activator of transcription
NF-κB: Nuclear factor kappa-light-chain-enhancer of activated B cells
IRF: Interferon regulatory factor
LDH: Lactate dehydrogenase
