# Supplementary material for: Human rhinovirus serotypes induces different immune responses
Source: Virol J. 2021 Nov 27;18:232. doi: 10.1186/s12985-021-01701-1 (PMC8626727; doi:10.1186/s12985-021-01701-1)
Supplement: Supplementary file 1 — Additional file 1. Figure S1. HRV16 RNA, LDH activity, and INF-λ1/3 and IL-6 levels after HRV16 infection in A549 cells. (a) The copy number of HRV16 was not different between 33°C and 37°C at 24 – 96 h post-infection. LDH activity also did not differ between 24, 48, 72, and 96 h post-infection. (c) INF-λ1/3 and IL-6 levels increased consistently from 24 to 72 h and remained unchanged from 72 to 96 h. Figure S2. Dose-response of HRV16 RNA, LDH activity, and IFN-β, IFN-λ1/3, and IL-6 production from submerged primary nasal epithelial cells after HRV16 infection. (a) At 72 h post-infection, HRV16 RNA was not different in nasal epithelial cells infected with 0.5 MOI and 1 MOI of HRV16. (b) At 24, 48, and 72 h post-infection, LDH activity and IFN-β, IFN-λ1/3, and IL-6 production were not different in nasal epithelial cells infected with 0.5 MOI and 1 MOI of HRV16. [file 12985_2021_1701_MOESM1_ESM.docx]

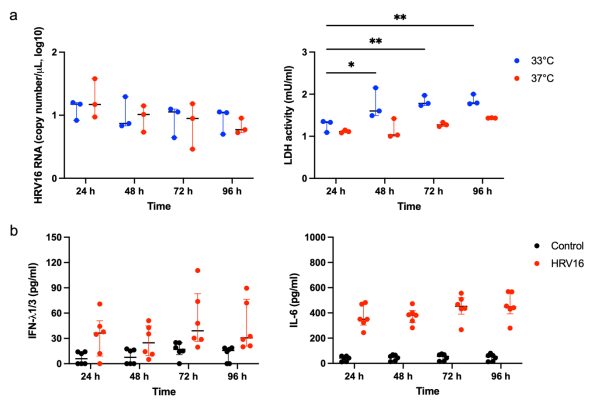


**Figure S1.** **HRV16 RNA, LDH activity, and INF-λ1/3 and IL-6 levels after HRV16 infection in A549 cells.** (a) The copy number of HRV16 was not different between 33°C and 37°C at 24 – 96 h post-infection. LDH activity also did not differ between 24, 48, 72, and 96 h post-infection. (c) INF-λ1/3 and IL-6 levels increased consistently from 24 to 72 h and remained unchanged from 72 to 96 h.


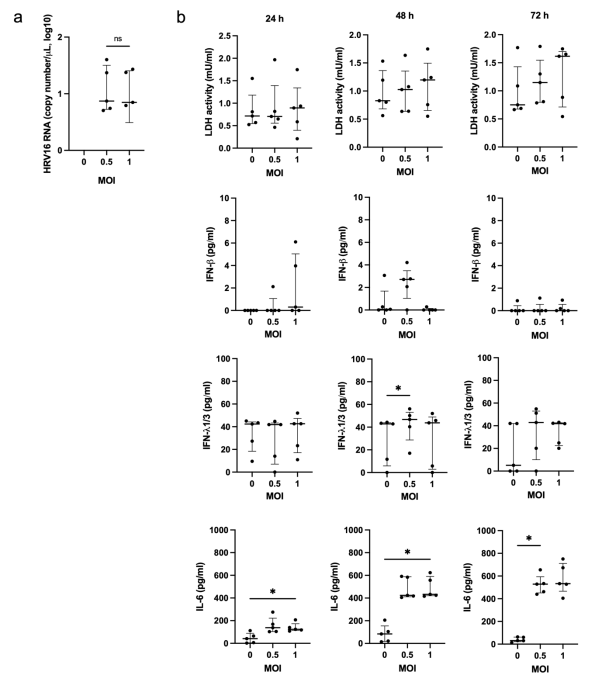


**Figure S2. Dose-response of HRV16 RNA, LDH activity, and IFN-β, IFN-λ1/3, and IL-6 production from submerged primary nasal epithelial cells after HRV16 infection.** (a) At 72 h post-infection, HRV16 RNA was not different in nasal epithelial cells infected with 0.5 MOI and 1 MOI of HRV16. (b) At 24, 48, and 72 h post-infection, LDH activity and IFN-β, IFN-λ1/3, and IL-6 production were not different in nasal epithelial cells infected with 0.5 MOI and 1 MOI of HRV16.
